# Surface Modification and Crystal Quality Improvement of 4H-SiC Film via Laser Treatment: Comparison of Continuous Wave and Femtosecond Pulse Laser

**DOI:** 10.3390/ma18081781

**Published:** 2025-04-14

**Authors:** Xu Han, Jiantao Zhou, Rui Li, Shizhao Wang, Fang Dong, Chengliang Sun, Sheng Liu

**Affiliations:** 1 The Institute of Technological Sciences, Wuhan University, Wuhan 430072, China; leo_han@whu.edu.cn (X.H.); zhou_jiantao@whu.edu.cn (J.Z.); rui_li@whu.edu.cn (R.L.); shizhao_wang@whu.edu.cn (S.W.); 2 Ningbo Institute of Materials Technology and Engineering, Chinese Academy of Sciences, Ningbo 315201, China; 3 Wuhan Institute of Quantum Technology, Wuhan 430206, China; 4 Hubei Key Laboratory of Electronic Manufacturing and Packaging Integration, Wuhan University, Wuhan 430072, China; 5 Key Laboratory of Transients in Hydraulic Machinery, Wuhan University, Ministry of Education, Wuhan 430072, China

**Keywords:** 4H-SiC, continuous wave laser, femtosecond pulse laser, laser treatment, surface defect, surface roughness

## Abstract

4H-SiC (silicon carbide), known as the third-generation semiconductor, has been widely used in high-power electronic devices. However, surface defects on wafers can seriously affect the key parameters and stability of silicon carbide devices. In this work, we pioneered a dual-laser comparative framework to systematically investigate the effects of continuous wave (CW) and femtosecond (FS) pulse laser micromachining on 4H-SiC epitaxial layers. CW laser restructuring optimized lattice integrity at sub-melting thresholds, while ultrafast FS pulse laser achieved submicron roughness control (from 8 μm to <0.5 μm) without obvious thermal collateral damage. To reveal the dynamic mechanism during the laser modification, multi-physics finite element models were adopted that decouple thermal and non-thermal mechanisms. This work expands the feasibility of laser micromachining for next-generation SiC device manufacturing.

## 1. Introduction

Silicon carbide (SiC) is a typical representative material of the third-generation semiconductors widely used for high-power electronic devices [1,2]. SiC-based devices are capable of working with high current density and high-temperature operation. These devices are fast-switching and simultaneously have low conduction/switch loss [3,4]. However, the key parameters of silicon carbide devices, including switching speed, threshold voltage, and stability, are severely affected by the interface defects present in the gate oxide structure or the region doped through ion implantation [5]. Various defects introduced during SiC crystal preparation and epitaxial growth threaten the reliability of SiC-based devices [6]. At the same time, subsurface damage induced in polishing and ion implantation results in surface defects [7]. Triangular defects in the active layers of p–n junction diodes have been reported to reduce the device breakdown voltage by more than 65% [8]. Moreover, carrot defects were reported to increase the reverse leakage current of 4H-SiC Schottky barrier diodes and p–n junction diodes [9]. Precise modification of 4H-SiC surface defects has become a critical bottleneck for advancing high-power electronics in electric vehicles and renewable energy systems.

Usually, thermal annealing and chemical mechanical polishing (CMP) are employed to modify SiC film quality. With conventional annealing methods (such as furnace and rapid thermal annealing), high temperature pervades the whole sample, resulting in obvious deformation and interlayer diffusion [10]. Additionally, due to the hard abrasives and corrosive slurries used, polished materials are prone to surface defects, such as scratches, dishing, and erosion during the CMP process [11]. Common polishing methods have the limitation of locally improving the surface roughness. The escalating demand for wide-bandgap semiconductors in high-power electronics and quantum devices necessitates defect-free 4H-SiC surfaces, and the rapid scaling of 4H-SiC wafer production further demands non-contact, site-selective processing to replace conventional CMP isotropic material loss. Laser processing has become an advanced non-contact surface machining technology with high precision, high efficiency, low pollution, and directional processing advantages. High flexibility and remarkable geometric accuracy of surface cleaning can be ensured [12]. The reduction in defects can be effectively achieved under laser irradiation. This method allows for a rapid escalation in heat velocity, an elevated thermal gradient, and a more controllable heating range, collectively contributing to the film surface’s cleaning [13,14]. It can focus on the defect regions and ensure increased film densification, decreased dislocation density, improved surface flatness, and improved crystal quality.

Continuous-wave or pulsed laser irradiation has been utilized to polish silica [15], diamond [16], and metals [17], eliminate shallow defects, and perform material stripping. On the one hand, the surface material can be melted via heat under the irradiation of the laser beam. Then, the surface flow is generated by surface tension and the Marangoni effect to produce morphological repair [18]. On the other hand, the surface material is ablated through strong absorption of laser photons to optimize the morphology. As for ultrafast lasers, ablation is caused by multi-photon absorption at high peak intensity so that even materials transparent to the laser wavelength can be machined [19]. Nevertheless, current research on laser annealing mainly concentrates on modifying the injection layer after ion implantation [20,21]. The direct repair of surface defects on SiC crystals is rarely reported [22]. Zheng et al. demonstrated the polishing of SiC ceramics using a high-frequency femtosecond laser, producing a pit-free SiC surface with an average roughness (Sa) of 0.187 μm, offering insights into effective defect removal in SiC ceramics [22]. Hecht et al. theoretically studied the influence of laser parameters on temperature and stress distribution and experimentally found the optimized polishing parameters to decrease the roughness of fused quartz [23]. Zhang et al. proposed in situ nitrogen doping with laser annealing to enhance the electrical performance of PECVD SiC, transforming it to polycrystalline form and improving carrier activity and conductivity, making it suitable for MEMS and post-CMOS applications [24].

This study compared the effects of CW and FS pulse lasers on the surface defects of the 4H-SiC epitaxial layer. The surface morphology and crystal quality for the laser-modified surface were assessed using scanning electron microscopy (SEM), X-ray diffraction (XRD), Raman spectra, energy dispersive X-ray spectroscopy (EDS), and 3D optical profiling. To reveal the interaction mechanism between different types of lasers and 4H-SiC, a multi-physics finite element method (FEM) simulation was conducted to examine temperature and flow evolutions.

## 2. Materials and Methods

### 2.1. Laser Treatment Procedures

The experimental sample was a 330 μm-thick 4H-SiC substrate (Orientation: [0001] ± 0.5°, Si face, Ra ≤ 0.2 nm, Resistivity: 0.02–0.1 Ω·cm) with a 50 μm n-type epitaxial layer. Each sample with a 2-inch diameter was divided into several areas for laser micromachining. Before the irradiation experiments, the 4H-SiC specimen was cleaned with acetone, ethyl alcohol, and deionized water for 10 min in an ultrasonic environment.

**Figure 1:**
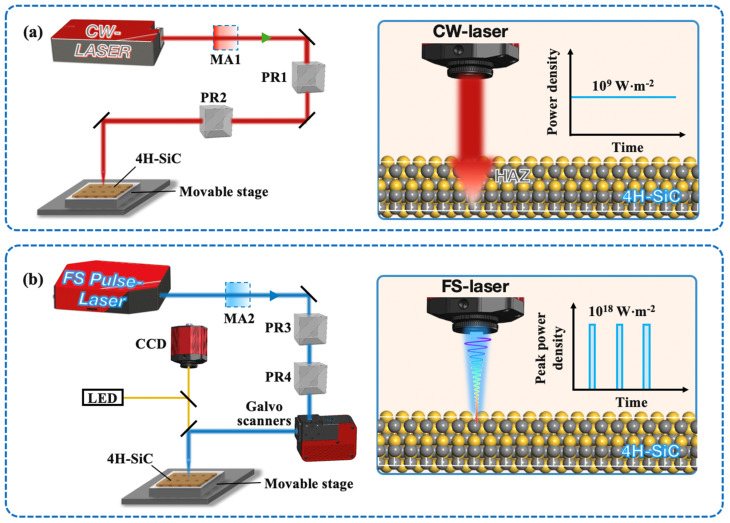
Schematic diagram of beam delivery unit laser irradiation. (**a**,**b**) Systems of various laser beams and interactions between the 4H-SiC surface and the beams, respectively.

Continuous-wave laser treatment:

A Yb-doped single-mode CW laser (YLR-500-AC; IPG Photonics Corporation, Oxford, MA, USA) was applied with a wavelength of 1050 nm and a spot size of about 240 μm. A schematic diagram of the experimental facility is given in Figure 1a. In the experiments, CW laser power ranging from 100 W to 200 W in power scanned the corresponding irradiated regions. After irradiation, the modified regions were characterized and compared with the defect-free region of the original sample.

Femtosecond pulsed laser treatment:

The surface of 4H-SiC epitaxial film was processed using 190 fs pulsed laser irradiation with a wavelength of 514 nm and a repetition rate of 1 MHz. The laser pulses were from a regenerative amplified Yb: KGW-based laser system (PHAROS SP-HP; Light Conversion UAB, Vilnius, Lithuania). The laser was focused with a microscope objective of 50×, yielding an in-focal-plane laser spot with a radius of about 0.5 μm. 4H-SiC films were mounted onto a nano-positioning system (ANTPLUS series), with movement accuracy of <0.25 μm in three axes. A schematic diagram of the optical setup is presented in Figure 1b. The average power of pulses varied from 80 mW to 140 mW, with a scanning speed of 0.1 mm/s. The pulsed laser was oriented horizontally with a distance between scanning lines of 0.5 μm to ensure the complete removal of the surface-protruding defects, maintaining a surface smoothness consistent with the defect-free region.

After laser treatment, the surface micromorphology and elemental composition of the samples were analyzed by emission scanning electron microscopy (SEM; MIRA 3; Tescan Ltd., Brno, Czech Republic) and energy-dispersive X-ray spectroscopy (EDS; X-Max 20; Oxford Instruments, Abingdon, UK). A 3D optical profiler (NewView 8000; ZYGO Ltd., Middlefield, OH, USA) was employed to detect the surface roughness. An X-ray diffractometer (XRD; SmartLab SE; Rigaku Corporation, Akishima, Japan) with a high-resolution setup was used to analyze crystal structure in the 2*θ* range 20–80° with Cu Kα. A Raman spectrometer (HR Evolution; HORIBA, Kyoto, Japan) was applied to measure crystalline quality and film stress. The Raman spectrometer is continuously illuminated with a 532 nm laser. The spectrum range is 80–2000 cm^−1^, with an objective of 100× and grating of 1800 (500 nm). The Raman spectrometer is calibrated with single-crystal silicon.

### 2.2. Modeling and Simulation

1. CW Laser Treatment of 4H-SiC

**Figure 2:**
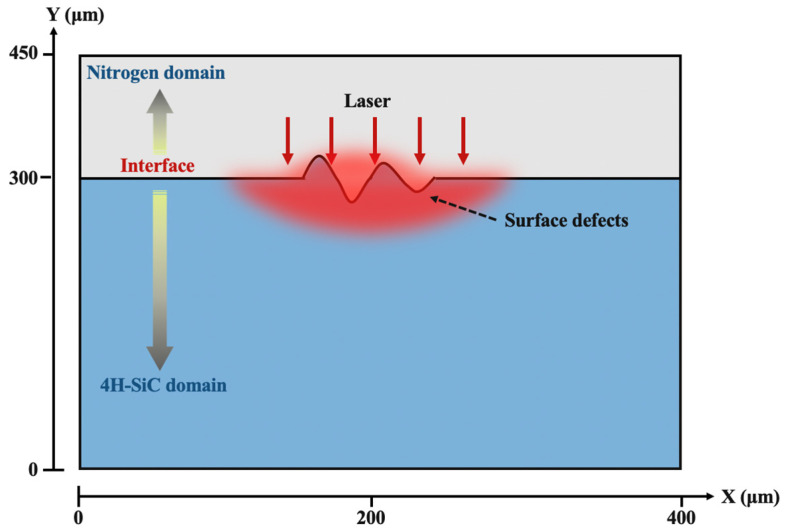
Schematic diagram of the 4H-SiC film model for laser irradiation.

To interpret the evolution mechanism of surface morphological defects during continuous wave laser irradiation, numerical simulation, including laminar flow, heat transfer, recoil pressure, gravity, surface tension, and Marangoni effect, was performed by COMSOL Multiphysics 6.0. The model employed the level-set method to monitor the geometric characteristics of the interface between the uneven surface and the atmospheric gas. Figure 2 shows a schematic diagram of the two-dimensional transient model with surface defect structures. The model spanned 400 µm in width and 300 µm in length, calibrating individual defect sizes to 20 µm, reflecting real-world conditions.

The motion of the flow phase in irradiated materials is described using the Navier–Stokes (N-S) equations, incorporating the source term as shown below:

(1) $$\rho (\frac{\partial u}{\partial t}+u\cdot \nabla u)=-\nabla [pI+\mu (\nabla u+{\left(\nabla u\right)}^{\tau })]+F$$

where ρ is the density of the material, u is the velocity, *p* is the flow pressure, *I* is the identity matrix, μ is the flow dynamic viscosity, and *F* is the source term to describe the interfacial forces, including gravity, surface tension, and Marangoni force.

Fourier’s law describes the relationship between heat flux density and temperature gradient in the process of heat conduction. It can be used to derive the heat conduction equation. The three-dimensional unsteady heat conduction equation includes a convection term when considering fluid velocity. This equation is known as the convection–diffusion equation, describing the temperature field’s variation with time and space in a fluid. It can be expressed as:

(2) $$\rho c_{P}(\frac{\partial T}{\partial t}+u\cdot \nabla T)=\nabla \cdot (k\nabla T)+q$$

where the *c*_p_ is the specific heat capacity, *T* and *t* are the temperature and time, respectively, *k* is the thermal conductivity, and *q* is the volumetric heat source term.

The thermal energy deposition of the laser beam was considered to follow a Gaussian distribution:

(3) $$Q_{\mathrm{laser}}=\frac{2\alpha P_{\mathrm{laser}}}{\pi r_{0}^{2}}\exp(\frac{-2{\left(x-x_{0}\right)}^{2}}{r_{0}^{2}})$$

where α is the absorptivity, the laser power *P*_laser_ is 150 W, the radius *r*_0_ of the laser spot at the focal point is 60 µm, and *x*_0_ is the initial position of the laser beam.

The Marangoni effect occurs when there are gradients in surface tension induced by temperature variation and can be expressed as:

(4) $$\sigma =\kappa \gamma \cdot n+\nabla_{s}\gamma$$

where *κ*, γ, and **n** represent the curvature, surface tension and interfacial normal vector, respectively. $\nabla_{s}\gamma$ denotes the surface thermal gradient. The material properties and processing parameters in this model are presented in Table 1.

2. FS pulse laser treatment of 4H-SiC

A two-dimensional transient model was further established to analyze the FS pulse laser energy deposition and propagation inside the 4H-SiC film. For the ultrashort-pulse (USP) laser, a two-temperature model (TTM) consisting of coupled ion and electronic systems was employed to describe and calculate electron and lattice temperature. The electron temperature *T*_e_ and lattice temperature *T*_l_ were calculated as follows:

(5) $$C_{e}\frac{\partial T_{e}}{\partial t}=\nabla (k_{e}\nabla T_{e})-G(T_{e}-T_{1})+S_{\mathrm{Laser}}$$

(6) $$C_{1}\frac{\partial T_{1}}{\partial t}=\nabla (k_{1}\nabla T_{1})+G(T_{e}-T_{1})$$

where *k*_e_, *C*_e_, *k*_l_, and *C*_l_ are the electronic thermal conductivity, heat capacity, lattice thermal conductivity, and heat capacity, respectively. *G* is the electron–lattice coupling coefficient.

**Figure 3:**
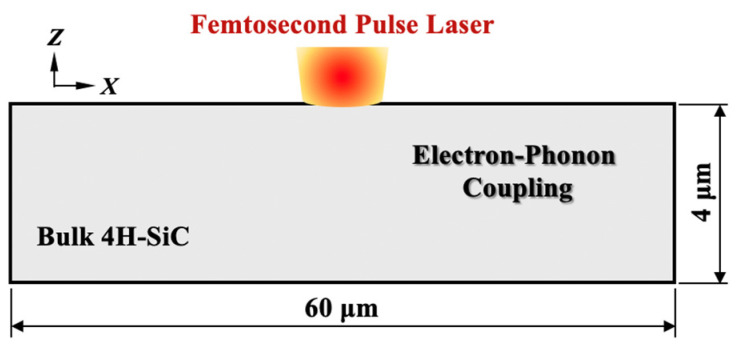
Schematic of femtosecond laser irradiations of 4H-SiC films.

The source term *S*_Laser_ can be numerically described as:

(7) $$S_{\mathrm{Laser}}=A\cdot I_{\mathrm{mod}}(x,t)\cdot \exp(Az)$$

where *A* is the laser energy absorption coefficient. The Gaussian laser intensity *I*_mod_(*x*,*t*) is numerically denoted as:

(8) $$I_{\mathrm{mod}}(x,t)=\frac{2E}{\pi \omega^{2}\tau }\cdot \exp\left[-4\ln2{\left(\frac{t-2\tau }{\tau }\right)}^{2}\right]\cdot \exp\left[-2{\left(\frac{x}{\omega }\right)}^{2}\right]$$

where *E* represents the laser energy, the pulse width *τ* is 120 fs, and the laser beam radius *ω* is 1 µm. The schematic of 4H-SiC film with FS pulse laser irradiation is illustrated in Figure 3.

## 3. Results and Discussion

### 3.1. Surface Morphology

**Figure 4:**
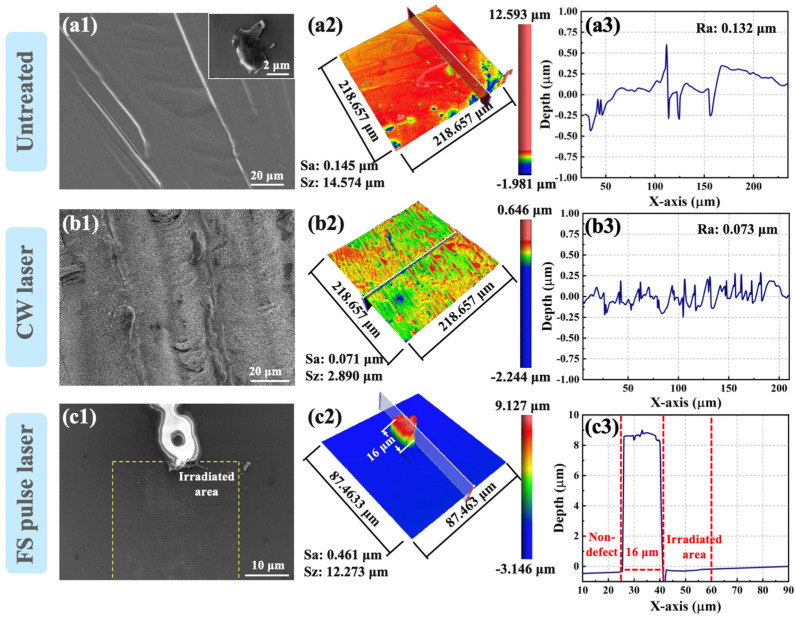
Micromorphology alterations induced by CW laser and FS laser treatment of surface defects in 4H-SiC. (**a1**–**a3**) the morphology of untreated 4H-SiC film; (**b1**–**b3**) the morphology of the CW laser-treated region; (**c1**–**c3**) the morphology of the region treated with FS pulse laser.

The surface topographies of 4H-SiC surface defects with and without laser treatment are revealed in Figure 4. Figure 4a represents the morphology of untreated 4H-SiC film. Figure 4b shows the morphology of the CW laser-treated region. Figure 4c shows the morphology of the region treated with the FS pulse laser. Continuous and uneven defects exist on the film surface in the SEM image of Figure 4(a1), and the inset represents another type of surface morphology with protrusions at the micrometer level. Figure 4(a2,a3) illustrate that the measured surface roughness (Sa) is 0.145 μm and the line roughness (Ra) is 0.132 μm. Concerning the laser treatment regions, a 150 W CW laser and a 100 mW FS pulsed laser were employed to selectively execute irradiation on the surface defects. In the SEM image of Figure 4(b1), the defect region of the film surface exhibits improved smoothness after CW laser treatment. However, wrinkle structures remain on the surface due to overlapping remelting regions. As depicted in Figure 4(b2,b3), the values of Sa and Ra are markedly reduced to 0.071 µm and 0.073 µm, respectively. Liu et al. explained a similar phenomenon when studying laser-assisted surface healing of AlN films. When the material is heated and melted by laser irradiation, due to the action of multiple forces, the convex parts flow downward along the surface, and the concave parts flow upward, making the surface smoother [28]. At the same time, Figure 4(c1) shows the SEM image on the micron scale after FS pulse laser treatment. It can be seen that the lower part of the defect is significantly eliminated. In addition, the maximum surface profile peak height of the selected defect reached 8 µm, as depicted in Figure 4(c2,c3), and there was no distinct difference in the roughness between the defect-free region and laser treatment region. The microscale surface smoothing and defect elimination are primarily attributed to the ultrafast non-thermal ablation and precise energy deposition characteristics of FS pulse laser processing [29,30]. The minor scale alterations of the irradiated region will be analyzed in the following discussion. Related research pointed out that the solid-plasma phase transition induced by the FS lasers leads to the formation of phase explosion accompanied by a shock wave and plasma expansion that can cause the surrounding material to crush [31].

### 3.2. Phase Changing

**Figure 5:**
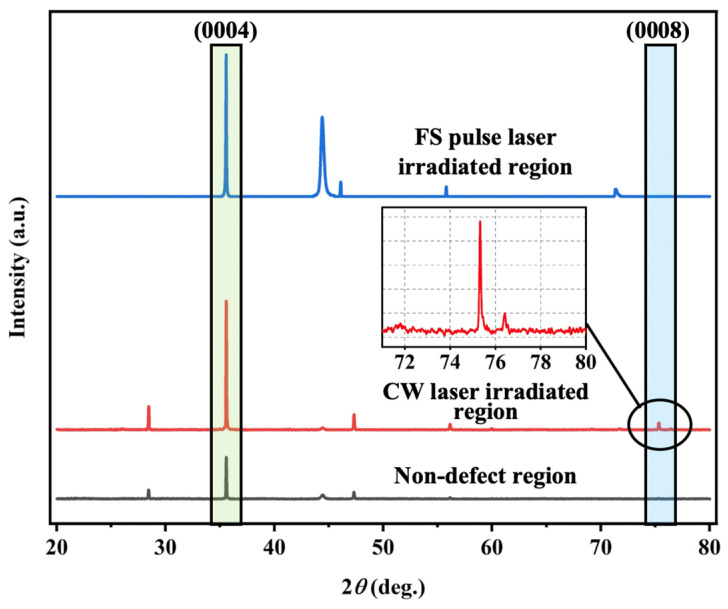
X-ray diffraction scans of different regions. The intensity of XRD peaks was normalized to the peak of the (0004) plane.

Figure 5 illustrates XRD patterns within the laser-affected regions. The XRD patterns exhibit a strong diffraction peak related to the (0004) crystal plane of 4H-SiC. Weak diffraction of the (0008) plane presents in defect-free and CW laser-treated regions. According to the extinction rule of crystal symmetry with space group P63mc for hexagonal SiC structure, only diffraction peaks from (000*l*) planes where *l* = 2*n* (*n* is an integer) will appear [32]. The peaks of the non-defect region and CW laser-treated region correspond to *l* = 4 and 8 or *n* = 2 and 4, denoting an excellent single crystalline structure with (0001) face in the normal direction of the measured regions. Delving into lattice information, Table 2 provides the position and strength of main peaks in three diverse regions. The positions of diffraction peaks in laser treatment regions barely shift compared to the non-defect region, indicating no apparent change in the lattice constant. This signifies the maintained crystal structure stability during the laser treatment. In Table 2, the rise in peak intensity related to the (0004) plane suggests improved structural order of the crystal lattice, indicating higher crystallinity. On the other hand, larger grains typically exhibit narrower and stronger diffraction peaks [33]. The FS laser-irradiated region demonstrates higher Raman peak intensity compared to the CW laser-treated area, attributed to the minimal thermal effects in FS laser processing. This preserves the superior crystallinity of the 4H-SiC lattice.

The crystallinity of the substrate was further evaluated by measuring the full width at half maximum (FWHM) of the omega scan rocking curve. The FWHM of the rocking curve is sensitive to lattice tilt and crystal bending, and can be expressed as a function of the dislocation density [34]. It is also a critical metric for quantifying crystallographic perfection. On the one hand, the FWHM reflects angular deviation (Δ*ω*), which is related to the local deviations in crystal orientation or lattice spacing. On the other hand, the FWHM directly correlates with enhanced crystalline coherence, reflecting long-range atomic order with minimal disruptions. Furthermore, residual stresses from processing or defects generate localized lattice distortions (Δ*d*/*d*), altering the effective Bragg angle across the sample. These variations manifest as a broader intensity distribution in the ω scan. Therefore, Table 2 summarizes the FWHM values corresponding to the main peaks diffracted from the (0004) plane. For the main diffraction peak corresponding to the CW laser-treated area, the FWHM value is 0.068, slightly larger than that of the non-defect region. The broadened peak can be attributed to the incompletely repaired defects. At the same time, the FWHM of the diffraction peak in the FS laser-treated area decreases to 0.047, implying more thorough defect repair and overall improvement in crystal quality. The above results concurrently affirm that the quality of the crystal is in high concordance with the surface morphology.

**Figure 6:**
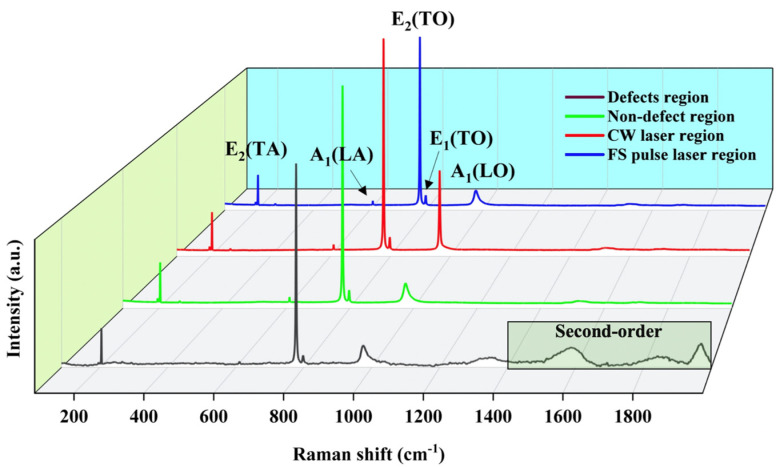
Raman survey spectra among various regions on 4H-SiC film.

Figure 6 depicts the Raman spectra generated by optical and acoustic phonons at a laser excitation wavelength of 532 nm of the different regions of the film. The Raman spectrum of the non-defect region is identifiable from previous studies, and the spatial symmetry of single 4H-SiC is $C_{6v}^{4}$, as presented in the spectrum [35]. In 4H-SiC, Raman-active modes (such as A_1_, E_1_, and E_2_ modes) are dictated by the $C_{6v}^{4}$ symmetry. These vibrational modes correspond to the motions of atoms that represent the crystal symmetry and are observed as peaks in the Raman spectrum. Figure 6 shows that the unique phonon mode E_2_(TA) is around 204 cm^−1^, and A_1_(LA), E_2_(TO), E_1_(TO), and A_1_(LO) are located around 610 cm^−1^, 777 cm^−1^, 798 cm^−1^, and 975 cm^−1^, respectively. However, the A_1_(LO) peak in the spectrum of the CW laser irradiation region shifted to 964 cm^−1^. The Raman shift is related to the density of free carriers [36]:

(9) $$n_{c}\;=\;1.25\;\times \;10^{17}\Delta \omega_{A1(\mathrm{LO})}$$

where *n*_c_ is the density of carriers and ∆*ω*_A1(LO)_ is the Raman shift of the A_1_(LO) mode in cm^−1^. The Raman peak of A_1_(LO) in the CW laser-irradiation area is redshifted toward the lower frequency due to the redistribution of carrier concentration inside the material caused by the remelting mechanism. During CW laser irradiation, the remelting process induces significant thermal effects that cause localized changes in the crystal lattice. This thermal input leads to a redistribution of carriers, which in turn alters the electronic environment and weakens the interatomic bonding forces. The reduction in bond stiffness and the possible expansion of the lattice result in a decrease in the vibrational frequency of the A_1_(LO) mode, manifesting as a redshift in the Raman spectrum (compared to the defect region).

The Raman peak of E_2_(TO) at 777 cm^−1^ shifting to a high wave number means that the material is under compressive stress. It moves to a low wave number when the material is under tensile stress. According to the biaxial stress model, the relationship [37] between silicon carbide stress and Raman shift is:

(10) $$\sigma_{\mathrm{res}}\;=\;-323\cdot \Delta \omega_{E2(\mathrm{TO})}$$

where *σ*_res_ is the residual stress and ∆*ω*_E2(TO)_ is the Raman shift of the E_2_(TO) mode.

Compared with the defect-free region of 4H-SiC, no specific 4H-SiC peak is found in the Raman shift among the laser treatment regions. The results indicate no tensile residual stress in the irradiated regions. Although the FS pulse laser interacts with 4H-SiC through phase explosion and plasma expansion extrusion, the precise occurrence of surface defects prevents residual stress formation [38]. For the CW laser, the remelting mechanism effectively eliminates the residual stress generated during the material surface processing.

After laser treatment, the Raman peaks are identical to the defect-free zone, maintaining the crystal structure of 4H-SiC. The disappearance of the Raman peak and the appearance of uplift in the second-order position suggest that the defect region undergoes an amorphous transformation in crystal. Along with the disappearance of the second-order peaks in the Raman spectra, this means that the repair of defects via laser treatment has suppressed the tendency of crystal-to-amorphous transformation in the region [26]. The intensity of the E_2_(TO) phonon mode at 777 cm^−1^ decreases from 3529.900 a.u. in the defect-free region to 3018.758 a.u. in the defect region. This denotes a decrease in the regularity of 4H-SiC. Nevertheless, both the laser-cleaned areas achieved a recovery in peak intensity and restoration of crystal uniformity. The FWHMs of folded transverse optical modes E_2_(TO) and E_1_(TO) reflect the discontinuity of the crystal structure. Table 3 summarizes the FWHM of FTO modes peaks. The increased FWHM observed in the CW laser-treated region, relative to both the defect-free and FS laser-treated areas, indicates that the remelting process partially eliminates the surface defect structures. As a result, the CW laser-treated region preserves some of the defect-induced microstructural features.

### 3.3. Laser Treatment Mechanisms

1. CW laser treatment of 4H-SiC

**Figure 7:**
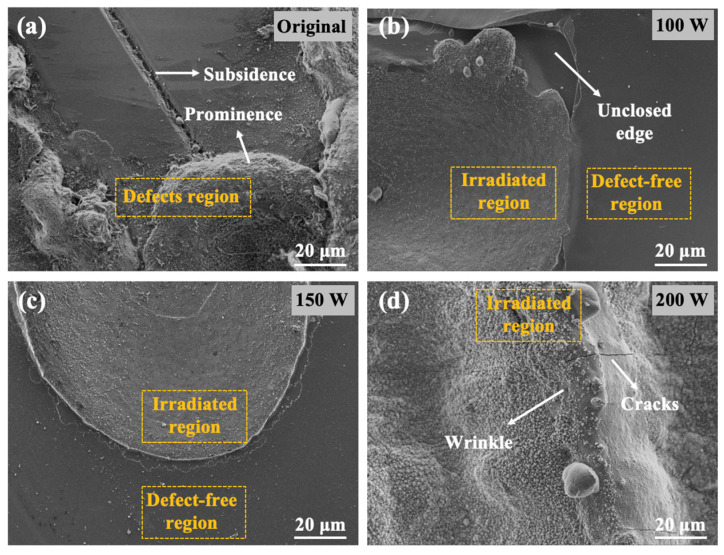
SEM images of 4H-SiC film surface morphology under CW laser irradiation. (**a**) Defect region of the original sample; (**b**–**d**) images of laser-irradiated film with 100 W, 150 W, and 200 W, respectively.

To elucidate the influence of CW laser irradiation on modified surfaces, the variation trends of surface defects on 4H-SiC under different power levels were investigated, as depicted in Figure 7. The defect regions exhibited continuous protrusions and depressions in the initial samples, as can be seen in Figure 7a. Obviously, the surface morphologies obtained at different laser powers display distinct variations. As shown in Figure 7b, the roughness of the central part of the defect region has been significantly improved. However, the edges exhibit unevenness and discontinuity. Figure 7c indicates that the morphology of the irradiated area at 150 W transformed into a relatively flat surface while seamlessly connecting with the adjacent non-irradiated area. Upon further increasing to 200 W, excessive energy and temperature caused severe damage to the surface structure, resulting in wrinkles and cracks that compromised the integrity of the crystal structure. These observations align closely with prior characterization results, suggesting a strong correlation between crystal quality and surface morphology.

**Figure 8:**
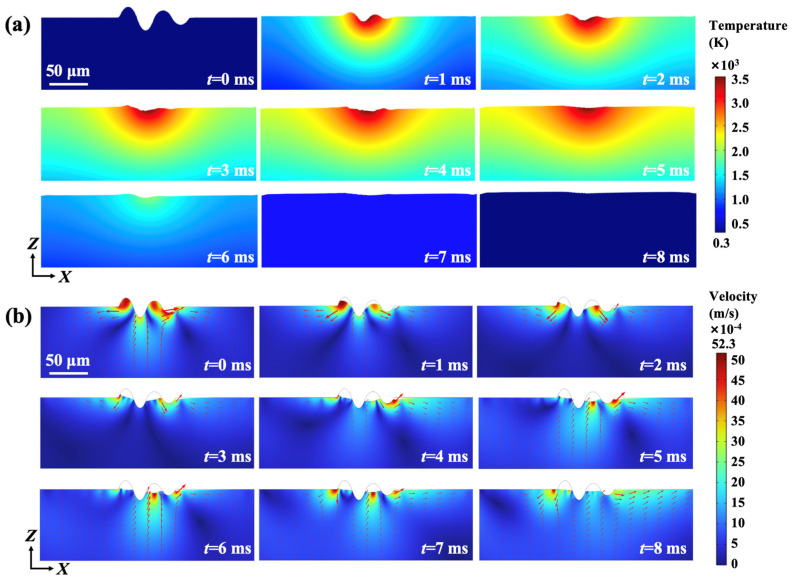
(**a**) Temperature distribution during CW laser irradiation; (**b**) velocity evaluation during CW laser irradiation.

The simulated temperature distribution during continuous wave laser irradiation is presented in Figure 8a. Under the heating of laser beam, the temperature of the upper surface increases and soon reaches the melting point of 4H-SiC. Subsequently, the molten pool and obvious temperature gradient occur and then expand rapidly, driven by the relatively stable heat source. In the molten pool, the melt flows from the center to the edge due to the negative surface tension coefficient [39]. Similarly to the drilling process, the shape and size of the depression appear significantly affected by the recoil pressure when the temperature exceeds the evaporation temperature [40]. The materials around the crest tend to flow into the trough under the combined effects of gravity, surface tension, Marangoni force, thermal buoyancy, and recoil pressure [41]. Therefore, the surface flatness is promoted as the laser continuously radiates the film. Chen et al. [42] also proved surface characteristic modification after laser polishing powder bed fused stainless-steel 316 L. Figure 8b depicts the flow velocity distribution in the molten pool during continuous wave laser irradiation, and the red arrows represent the fluid direction. The high velocity concentrates around the crests and the maximum velocity exceeds 5 mm/s. In the uneven area, two symmetrical vortices appear in the vertical direction and play significant roles in the mass transfer and surface modification [43]. As the surface material with low surface tension of the molten pool migrates from the inside to the outside, the velocity concentration is obviously alleviated under the Marangoni effect. As also mentioned by Zhao et al. [44], the recoil pressure is also of great importance to the flow velocity in the molten pool.

2. FS pulse laser defect ablation

**Figure 9:**
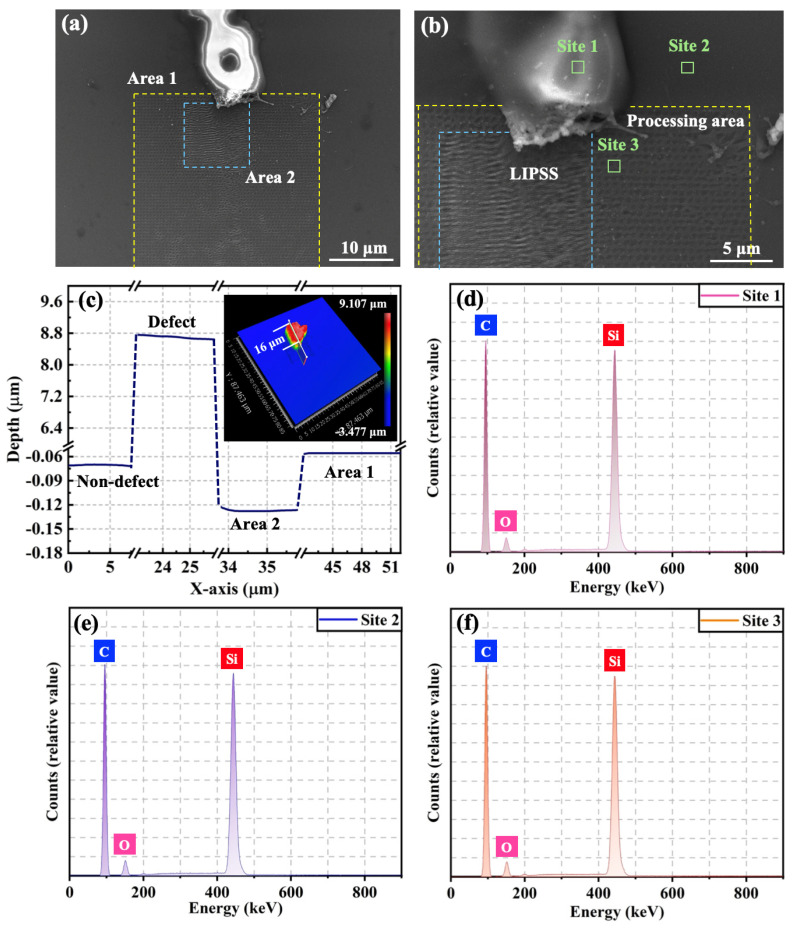
Surface morphology and EDS patterns of selected areas of 4H-SiC film. (**a**,**b**) SEM image of unprocessed defect area and enlarged image of the processed areas; (**c**) depth distribution curve along the line of the inset of optical morphology; (**d**–**f**) EDS patterns of surface defect, non-defect region, and irradiated region, respectively.

Figure 9a shows the SEM image of the defect area, where Area 1 depicts the area scanned by the FS pulse laser once and Area 2 represents the area subjected to two consecutive scans. The FS laser selectively targeted the lower half of the protruding surface defects, allowing for a direct comparison of the roughness between the laser-treated and unirradiated defect areas. Figure 9b is the magnified SEM image, which clearly shows that the FS pulse laser effectively removed the surface defect with the bugle structure. Additionally, the surface morphology in Area 1 exhibits a pulsed, spot-like pattern. In contrast, Area 2 displays laser-induced periodic surface structures (LIPSSs) resulting from the FS laser irradiation on the 4H-SiC film, which breaks the chemical bonds in SiC via electronic excitation and reduces the atomic density. Simultaneously, the ripples observed on the 4H-SiC film are induced by the FS laser irradiation due to the self-organization of the defect concentration field [45]. Figure 9c denotes the surface roughness image given by the optical profiler, which signifies that the height of the protruding part of the surface morphological defect can reach about 8.8 μm. In contrast, the surface roughness of the area after the pulse laser scanning machining is reduced to 0.015 μm. At the same time, the processing depth of Area 2 slightly increases compared with that of Area 1, but the surface flatness of the irradiated areas remains highly aligned with the defect-free area. The surface roughness of the irradiated regions demonstrates a significantly high-quality refinement.

EDS analysis was performed (Figure 9a) to obtain the element distribution of the selected laser-irradiated regions. Site 1 corresponds to the surface defect area, Site 2 represents the defect-free area, and Site 3 is the modified area where the laser removes the defect. Figure 9d–f depict the EDS spectrum and elemental distribution maps. The spectra suggest that the elemental distributions in the three regions are consistent, and the main components are Si and C elements. This proves that the protruding structure is a surface defect of 4H-SiC rather than oxide or other impurities introduced in the preparation or machining process. Table 4 summarizes the percentage of the weight content for each element in the EDS spectrum, which demonstrates that no change in properties occurs in the material after optimization by the FS pulse laser. This avoids severe thermal effects in the procedure of surface defect repair or material removal.

**Figure 10:**
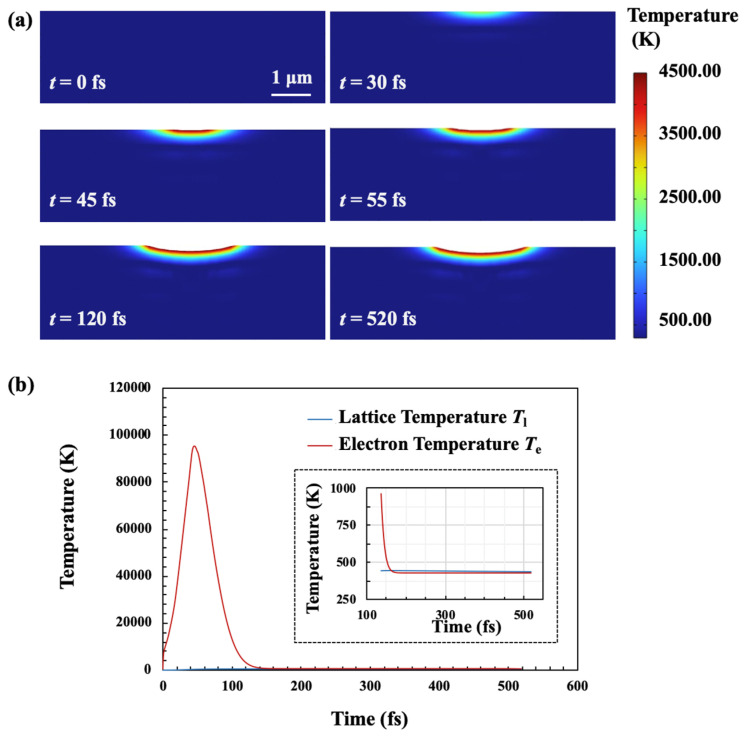
Temperature profiles during femtosecond laser pulse irradiation. (**a**) Temperature distribution on 4H-SiC during FS pulse laser irradiation; (**b**) electron and lattice temperature evolutions of FS pulse laser focus.

As depicted in Figure 10a, the simulation of the temperature distribution on a 4H-SiC film during FS pulse laser irradiation shows how the temperature evolves. Initially, no significant temperature rise is observed, but a localized heating area begins to appear by *t* = 30 fs. As time progresses, the temperature gradient becomes more pronounced, peaking at around 4500 K near the surface region, as shown by the red areas. Then, the heat continues to spread, but remains concentrated near the irradiated zone, highlighting localized heating effects under femtosecond laser pulses. Figure 10b shows the electron temperature and lattice temperature in the 4H-SiC. Due to the ultrahigh-power density of the FS pulse laser, the electron temperature rapidly increases to its peak (9.8 × 10^4^ K) at half the pulse duration and then swiftly decreases. The lattice temperature maintains equilibrium with insufficient time for any significant increase throughout the procedure. In ultrashort pulse lasers, energy absorption occurs on a much shorter timescale than its transfer to the lattice, effectively decoupling the absorption processes from lattice heating. Electrons in the conduction band are rapidly energized by the laser pulse, a process that outpaces the electrons cooled via phonon emission. As electron density increases through avalanche ionization, the plasma frequency approaches that of the incident laser radiation [46,47]. The solid-to-plasma phase transition induces the formation of nanovoids, accompanied by a shock wave and plasma expansion that generate extreme pressure inside the material, ultimately causing the surrounding material to crush [48].

### 3.4. Comparison of CW and FS Pulse Laser Modification

**Figure 11:**
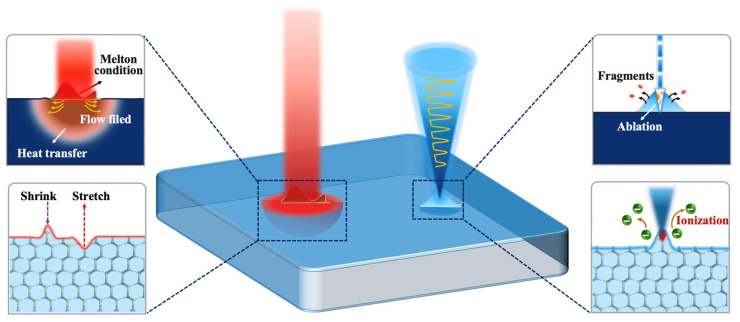
Schematic diagram of laser treatment mechanisms.

The above findings delineate that the CW laser and the FS pulse laser follow different mechanisms in treatment surface defects of the 4H-SiC, as depicted in Figure 11. In the case of the CW laser, energy is continuously input on the 4H-SiC film, culminating in material melting and a consequent resolidification profile. As schematized in the left part of Figure 11, FEM simulation revealed the role of temperature and flow field during the formation of the modified surface. A non-uniform temperature distribution produced the molten pool, and the recoil force provided by the vapor depended on the molten pool. An extension provided by the middle flow resulted in the widening of the molten pool. The middle flow also produced large shear stress for the liquid phase on the surface of the molten pool. When the shear stress exceeded a critical value, the topology of the molten pool changed, yielding the spatter. These results indicate that the CW laser is a thermally dominated melting process where the heat proliferates from the laser’s focal point to the encompassing region. Because CW laser treatment relies mostly on the melting, flow, and shrinkage of the molten pool, it causes heavy thermal effects in the heat-affected zones. During the melting process, there is no obvious material loss. Therefore, the interaction mechanism of the CW laser with the 4H-SiC material is more suitable for complex surfaces with defects in both protrusions and grooves.

Notably, the FS pulse laser exerts a less detrimental impact on the microstructure than the CW laser. This divergence can be traced back to the distinct interplay between the FS pulse laser and 4H-SiC film. Typically, the FS pulse laser generates an exceptionally high peak power density, reaching 10^18^ W/m^2^, which leads to matter loss through fragmentation spatter [49], as illustrated on the right of Figure 11. This effect arises from the short-pulse laser irradiated on the 4H-SiC surface, which injects energy into the electronic systems, forming a steep thermal gradient and elevated electronic temperature near the surface. The high electronic temperature gradient yielded a high electronic pressure to act on ions [50], leading to the lattice stretch along with the irradiation with direction. The high-temperature difference between the ions and the electrons resulted in energy exchange. After laser irradiation, the energy exchange was able to remain at (80–220) fs less than the irradiation time [51]. The ions were able to acquire additional kinetic energy from the electronic systems, with elevated kinetic energy and electron pressure ultimately driving near-surface ions to escape from the bulk 4H-SiC. These escaping ions achieve maximum kinetic energy exceeding 2 × 10^5^ eV, dispersing swiftly into the ambient environment and expelling a sizable cluster of fragments at a velocity approaching 200 m/s [51]. Thus, the rapid expulsion of defect fragments significantly precedes the heat diffusion into the adjacent material, curtailing the extent of the heat-affected zone. Given its mechanism of action, the femtosecond pulse laser is particularly well suited for treating surfaces with protruding defects or residual foreign material.

Although CW laser processing excels in repairing complex surfaces with interconnected protrusions and grooves, its thermal dominance imposes both advantages and limitations. Non-ablative melting enables seamless resolidification with minimal material loss, which is ideal for shallow-defect healing. However, residual thermoelastic stress caused by thermal effects may cause microcracks in high-power device applications. Conversely, the FS pulse laser achieves submicron defect selectivity via non-thermal fragmentation, but its efficacy is governed by defect geometry. Ultrafast spallation (200 m/s ejection velocity) eliminates protruding contaminants (such as carrot defects) with negligible HAZ, critical for preserving bulk crystallinity in high-frequency devices. However, its efficacy on subsurface defects (such as ion implant damage) is limited due to energy confinement within the electronic subsystem.

## 4. Conclusions

This study accentuates the feasibility and superiority of laser treatment as a leading-edge method for eliminating surface defects in 4H-SiC films. It also shows that CW and FS pulse lasers follow different mechanisms in removing surface defects by combining the characterizations and analysis of crystal quality and structure with simulation methods. The main conclusions are as follows.

(1) Under the optimized process parameters, both CW and FS pulse lasers significantly improve the morphology of the defect region and greatly reduce the surface roughness. The CW laser reduces the surface roughness to less than 0.071 µm, while the FS pulse laser can control the maximum surface profile peak height to about 0.015 µm. Laser treatment maintains the structural integrity of 4H-SiC and enhances overall quality according to XRD patterns.
(2) The CW laser remelting mechanism partially retains the structure caused by surface defects. In contrast, the FS pulse laser removes defect structures more thoroughly and can improve crystal quality more effectively. Benefiting from the ultrashort pulse duration and high-energy focal region, the irradiated area exhibits minimal thermal diffusion.
(3) Combined with FEM simulation, this reveals that the upper-surface temperature of the 4H-SiC crystal elevates rapidly to the melting point under the heating of the CW laser beam. The material flows from the crest to the trough, largely ascribed to the surface tension and Marangoni force, improving surface flatness. With FS pulse laser irradiation, rapid electron heating occurs via localized thermal effects. Under such a mechanism, the FS pulse laser eliminates surface defects at the microlevel.
(4) In general, the CW laser is suitable for processing complex surfaces with defects in both protrusions and grooves. The FS pulse laser is particularly well suited for treating surfaces with protruding defects or residual material.

While CW and FS lasers demonstrate distinct advantages, their efficacy is constrained by defect depth and geometry. CW laser-induced thermal stresses need to be considered, and FS pulse laser ablation shows limited efficacy on subsurface defects due to energy confinement. Additionally, this study focused on 1030 nm-wavelength regimes, but broader spectral optimization (such as with UV-FS lasers) remains unexplored.

Consequently, laser treatment represents a highly efficient method to modify surface defects and crystal quality. Future work will focus on laser treatment of the electrical properties of 4H-SiC.

## Figures and Tables

**Table 1:** Material properties and global expressions in temperature field simulation [25,26,27].

Parameters (Unit)	Symbol	Value
Melting temperature (K)	*T*_melt_	2973
Initial temperature (K)	*T*_0_	293.15
Thermal conductivity (W·m^−1^·K^−1^)	*k*	2.67 × 10^5^*T*^−1.26^
Solid specific heat (J·g^−1^·K^−1^)	*c*_p_	0.48 + 0.0023exp(*T*/262)
Solid density (kg·m^−3^)	*ρ*_s_	3210
Latent heat of fusion (J·kg^−1^)	*L*_m_	3.6 × 10^5^
Emissivity	*ε*	0.8 − 0.4exp(−*T*/600)
Absorption coefficient (m^−1^)	*α*	7.3 × 10^7^
Reflectivity	*R*	0.224
Electron specific heat capacity (J·m^−3^·K^−1^)	*C*_e_	78.300
Lattice specific heat capacity (J·m^−3^·K^−1^)	*C*_l_	1.659 × 10^5^
Electronic thermal conductivity (W·m^−1^·K^−1^)	*k*_e_	27.300
Lattice thermal conductivity (W·m^−1^·K^−1^)	*k*_l_	1.480
Electron–lattice coupling coefficient	*G*	1.120 × 10^17^

**Table 2:** Intensity and full width at half maximum (FWHM) of peak from the (0004) plane of the XRD pattern.

Measured Region	Omega (°)	Intensity (a.u.)	FWHM (°)
Non-defect region	35.581	1310	0.053
CW laser-irradiated region	35.540	4049	0.068
FS pulse laser-irradiated region	35.569	4960	0.047

**Table 3:** FWHM values of FTO modes.

Measured Region	E_2_(TO) (Raman Peak at 777 cm^−1^)	E_1_(TO) (Raman Peak at 798 cm^−1^)
Defect region	2.43 ± 0.05	2.86 ± 1.31
Non-defect region	2.38 ± 0.02	1.85 ± 0.35
CW laser treatment region	2.43 ± 0.02	2.48 ± 0.37
FS pulse laser treatment region	2.42 ± 0.01	2.31 ± 0.40

**Table 4:** Elemental composition of the selected regions of the 4H-SiC film (in wt. %).

Elements	Spectrum 1	Spectrum 2	Spectrum 3
	wt. %	wt. %	wt. %
Si	53.58	54.32	52.92
C	37.10	39.91	38.36
O	9.31	5.77	8.72

## Data Availability

The original contributions presented in this study are included in the article. Further inquiries can be directed to the corresponding authors.
